# Deep Learning-Enabled Diagnosis of Abdominal Aortic Aneurysm Using Pulse Volume Recording Waveforms: An In Silico Study

**DOI:** 10.3390/s25216678

**Published:** 2025-11-01

**Authors:** Sina Masoumi Shahrbabak, Byeng Dong Youn, Hao-Min Cheng, Chen-Huan Chen, Shih-Hsien Sung, Ramakrishna Mukkamala, Jin-Oh Hahn

**Affiliations:** 1 Mechanical Engineering, University of Maryland, College Park, MD 20742, USA; smasoumi@umd.edu; 2 ONEPREDICT Inc., Seoul 06160, Republic of Korea; bdyoun@snu.ac.kr; 3 Mechanical Engineering, Seoul National University, Seoul 08826, Republic of Korea; 4 Department of Internal Medicine, National Yang-Ming University, Taipei 112304, Taiwan; hmcheng@vghtpe.gov.tw (H.-M.C.); chench@vghtpe.gov.tw (C.-H.C.); 5 Institute of Emergency and Critical Care Medicine, National Yang-Ming University, Taipei 112304, Taiwan; mr.sungsh@gmail.com; 6 Bioengineering and Anesthesiology and Perioperative Medicine, University of Pittsburgh, Pittsburgh, PA 15261, USA; rmukkamala@pitt.edu

**Keywords:** abdominal aortic aneurysm, point-of-care diagnostics, machine learning, deep learning, arterial pulse waveform, pulse volume recording

## Abstract

This paper investigates the feasibility of diagnosing abdominal aortic aneurysm (AAA) via deep learning (DL)-enabled analysis of non-invasive arterial pulse waveform signals. We generated arterial blood pressure (BP) and pulse volume recording (PVR) waveform signals across a diverse synthetic patient cohort using a systemic arterial circulation model coupled with a viscoelastic model relating arterial BP to PVR while simulating a range of AAA severity levels. We confirmed the plausibility of the synthetic data by comparing the alterations in the simulated waveform signals due to AAA against previously reported in vivo findings. Then, we developed a convolutional neural network (CNN) with continuous property-adversarial regularization that can estimate AAA severity from brachial and tibial PVR signals. We evaluated the algorithm’s performance in comparison with an identical CNN trained on invasive arterial BP waveform signals. The DL-enabled PVR-based algorithm achieved robust AAA detection across different severity thresholds with area under the ROC curve values >0.89, and showed reasonable accuracy in severity estimation, though slightly lower than its invasive BP counterpart (MAE: 12.6% vs. 10.3%). These findings suggest that DL-enabled analysis of PVR waveform signals offers a non-invasive and cost-effective approach for AAA diagnosis, potentially enabling accessible screening through operator-agnostic and point-of-care technologies.

## 1. Introduction

Abdominal aortic aneurysm (AAA) is a potentially life-threatening localized dilation in the abdominal aorta, part of the main artery supplying blood to the body, generally defined as an increase in diameter of more than 50% relative to the artery’s expected normal size [1]. In clinical practice, a fixed threshold of 3.0 cm is commonly used to define an AAA, corresponding to more than two standard deviations above the mean aortic diameter in men [1]. Worldwide, about 1.5% of men between 50 and 60 years of age have AAAs >3.0 cm in diameter, and the prevalence of such clinically significant AAAs increases by 2–3% per decade thereafter [2]. Although AAA incidence may have declined in recent years due to, e.g., the decreasing prevalence of smoking in high-income countries, its future occurrence may rise substantially as the population ages [2]. AAA is subject to the risk of rupture due to the vessel expansion caused by weakened aortic wall and the distending blood pressure (BP) [3]. AAA rupture carries 85% mortality, ranking it among the United States’ ten leading causes of death in men older than 55 years [4]. In women, AAAs are 4–6 times less common [2,5] but experience more rapid growth and are 4 times more likely to rupture [5]. While elective surgical repair of AAA, open or endovascular, carries a 1-year mortality of about 6%, repair performed after rupture is associated with a nearly seven-fold higher 1-year mortality of approximately 40% [6,7]. Because most AAAs remain asymptomatic until rupture, effective screening and surveillance are essential to enable timely intervention.

Definitive diagnosis and surveillance of AAAs hinge on imaging methods operated by an expert, including ultrasound, CT, and MRI. Ultrasound is preferred due to its high sensitivity (94–100%), high specificity (98–100%), safety, and relatively low cost [8]. The US Preventive Services Task Force (USPSTF) recommends one-time ultrasound screening in men aged 65–75 years who have ever smoked [8]. Nevertheless, AAAs are often incidentally found and diagnosed during imaging for other conditions because screening and surveillance remain underutilized. Based on a recent study, more than 60% of privately insured patients in the US do not receive the recommended screening, with the gap especially pronounced in non-metropolitan areas [9]. Barriers include limited awareness of the asymptomatic nature of AAA, the inconvenience of attending in-person appointments, and associated costs [10]. As a result, operator-agnostic convenient technologies that can supplement ultrasound to support more frequent and convenient AAA screening are desired. In particular, technologies compatible with routine cuff-based measurements can open up unprecedented opportunities for population-level pre-ultrasound AAA screening and post-surgical AAA monitoring in home, primary care, and point-of-care settings.

Noting that AAA alters aortic geometry and compliance and thereby leaves measurable imprints on upstream and downstream arterial blood pressure and flow waveforms, physiological waveform analysis may offer a potential solution for convenient AAA screening. The arterial BP waveform results from the superposition of forward- and backward-traveling arterial BP waves that pass by the measurement location [11]. In the presence of AAA, local increase in vessel diameter and stiffness [12] alters both (i) local wave speed and (ii) characteristic impedance. They collectively modify normal wave propagation and waveform morphology, while the latter creates a site reflecting some of the wave energy and transmitting the rest. These changes manifest as a decrease in aortic pulse wave velocity (PWV) and an increase in undulations in arterial BP waveforms due to enhanced wave reflections [13]. Indeed, PWV was lower in AAA patients compared to age-matched controls [14], and undulations in both proximal and distal arterial BP waveforms disappeared after aneurysm repair [13]. Together, these observations support the potential of arterial pulse waveform analysis for AAA detection. Recent research has begun to exploit this promise, but existing approaches have important limitations: many require multiple invasive measurements [15,16], which are costly and impractical, or rely on photoplethysmography (PPG) [17,18], which is sensitive to noise and peripheral vasomotor tone, and may not capture sufficient hemodynamic information to identify subtle aneurysm-induced changes. Consequently, there remains a critical need to develop affordable, convenient, and non-invasive arterial pulse waveform-based methods for AAA diagnosis.

To bridge the gap, we investigated the feasibility of AAA diagnosis based on the analysis of non-invasive arterial pulse waveform signals called pulse volume recording (PVR) signals, which can be easily measured using low-cost BP cuff devices. Compared with ultrasound, PVR-based AAA diagnosis may be associated with lower cost and operator dependency. For instance, among commercially insured patients in the United States, the median price of a primary care office visit was about $60 [19], whereas a large hospital survey reported a median price of $532 for an abdominal ultrasound [20]. In addition, compared with the recently emerging PPG-based AAA diagnosis, PVR-based AAA diagnosis may be associated with higher accuracy and robustness (now that PVR signals are measured at relatively more proximal locations than PPG and are thus less prone to confounding factors such as peripheral vasomotor tone and environmental factors, e.g., skin color and ambient lighting condition). We used a 1-dimensional mathematical model of systemic arterial circulation and a viscoelastic model relating arterial BP to PVR to create arterial pulse waveform signals corresponding to a physiologically diverse synthetic cohort of healthy individuals and AAA patients. We evaluated the plausibility of the synthetic data using the findings of prior in silico and in vivo studies. We then developed a deep learning (DL)-enabled algorithm equipped with continuous property-adversarial regularization (CPAR), which can estimate AAA severity by analyzing brachial and tibial PVR waveform signals, and finally, assessed its performance in comparison with the same algorithm using invasive arterial BP waveform signals.

This paper is organized as follows. Section 2 describes the mathematical model for the generation of physiologically plausible synthetic arterial BP and PVR waveform signals, the structure and the training method of the DL-enabled AAA diagnosis algorithm, and the performance evaluation approach. Section 3 presents the results of the plausibility analysis of the synthetic data and the performance of the DL-enabled AAA diagnosis algorithm, which are discussed in Section 4. Section 5 concludes the paper.

## 2. Materials and Methods

### 2.1. Data Generation

**Figure 1:**
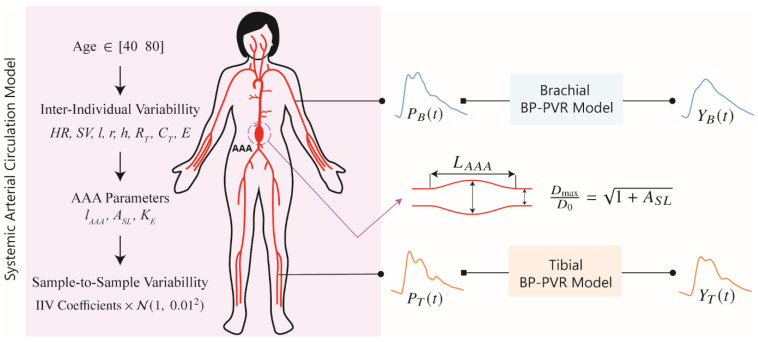
Mathematical model for the generation of physiologically plausible arterial blood pressure (BP) waveform and pulse volume recording (PVR) signals. The mathematical model is composed of a multi-branch transmission line (TL) model of systemic arterial circulation and viscoelastic models relating arterial BP to PVR at brachial and tibial arteries. *HR*: heart rate. *SV*: stroke volume. *l*: length of a TL. *r*: radius of a TL. *h*: thickness of a TL. *R_T_*: terminal resistance of a peripheral load. *C_T_*: terminal compliance of a peripheral load. *E*: Young’s modulus of a TL. AAA: abdominal aortic aneurysm. *l_AAA_*: ratio of AAA length to the length of TL_31_. TL_31_: TL pertaining to the abdominal aorta between right renal artery and inferior mesenteric artery. *A_SL_*: relative increase in maximum luminal area. *K_E_*: scaling coefficient for Young’s modulus pertaining to AAA wall. *P_B_*: brachial BP. *P_T_*: tibial BP. *Y_B_*: brachial PVR. *Y_T_*: Tibial PVR. IIV: inter-individual variability. N: normal distribution.

To develop and evaluate a DL-enabled arterial pulse wave analysis algorithm for non-invasive diagnosis of AAA based on PVR, we generated a large number of physiologically plausible arterial pulse waveform signals (including arterial BP and PVR) pertaining to a wide range of AAA severity levels using a mathematical model of systemic arterial circulation and viscoelastic models relating arterial BP to PVR at brachial and tibial arteries (Figure 1).

The data generation process is explained in detail in the next sections and is summarized here (Figure 1):

1. A cohort of synthetic patients between 40 and 80 years old with age-specific cardiovascular parameters is initialized.
2. Each patient is made unique by varying the parameters in the systemic arterial circulation model using inter-individual variability coefficients.
3. AAA is introduced in each subject.
4. Arterial BP waveform samples corresponding to each subject are generated while including sample-to-sample variability.
5. In each patient, the parameters in the viscoelastic model are sampled from previously obtained distributions.
6. PVR waveform samples corresponding to the arterial BP waveform samples are generated while incorporating sample-to-sample variability in the BP-PVR model.

#### 2.1.1. Generation of Arterial BP Waveform Signals

We generated a large number of synthetic arterial BP waveform signals (called “arterial BP waveform signals” hereafter) using a mathematical model of systemic arterial circulation developed in a prior work [21,22], which can simulate BP and blood flow wave propagation and reflection in 55 arteries in the arterial tree. It consists of 55 uniform and linear transmission lines (TLs), each representing an artery characterized by its geometric, viscous, elastic, and inertial properties (see Table S1). Most distal arteries are connected to 3-parameter Windkessel models representing small arterioles and capillaries. The systemic arterial circulation model simulates BP waveforms at all the 55 arteries when an aortic blood flow waveform is inputted to the ascending aorta. The systemic arterial circulation model was validated against physiological measurements and the results of other studies [22]. In addition, it was successfully used in prior investigations of arterial viscoelasticity, arterial stenosis, and peripheral artery disease diagnosis [21,23,24].

Using the systemic arterial circulation model, we generated arterial BP waveform signals corresponding to a cohort of synthetic patients (called “patients” hereafter) according to the framework below (Figure 1):

First, we specified the patient’s age from the range of 40 to 80 years as a key covariate in our systemic arterial circulation model to incorporate the age dependence of the nominal values pertaining to stroke volume, arterial geometry, vascular compliance, peripheral resistance, and arterial stiffness. According to the formulas reported in [25] (which are presented in the Supplementary Material), an older age is associated with lower stroke volume, longer proximal aorta (up to the aortic arch), larger arterial diameters, reduced vascular compliance, and increased peripheral resistance and arterial stiffness.

Second, to introduce inter-individual variability (IIV), the values of systemic arterial circulation model parameters in a patient were assigned by multiplying their nominal values by IIV coefficients randomly sampled from uniform distributions specific to individual parameters. These coefficients ranged from 0.8 to 1.2 for heart rate (60–90 bpm), stroke volume, TL lengths, radii, and thicknesses, terminal resistances, and terminal compliances, and from 0.7 to 1.6 for arterial stiffness (corresponding to 5–15 m/s carotid–femoral PWV). This enabled us to have a diverse patient cohort while maintaining physiological plausibility.

Third, AAA was introduced in each patient by modifying their sub-renal abdominal aorta (TL_31_). The AAA length $l_{AAA}$ was set between 40% and 100% of the original length of TL_31,_ reflecting the range of AAA sac heights reported in [26]. Then, the stiffness of the AAA region was increased by a factor $K_{E}$ ranging from 1.25 to 2.45, reflecting the interquartile range (IQR) of AAA-to-remote aortic stiffness ratios reported in [12]. Subsequently, the AAA radius was defined along its length using Equation (1) [16]:

(1) $$r\left(x\right)=\;r_{0}\sqrt{\left(1\;+\frac{A_{SL}}{2}\right)-\;\left(\frac{A_{SL}}{2}\right)\times \;cos\left(2\pi \left(x\;+\;0.5\right)\right)},\;-0.5\leq x\leq 0.5$$

where x is the normalized position along the AAA length (x=0 at the AAA center), $r_{0}$ is the pre-AAA diameter, and $A_{SL}$ is the relative increase in maximum AAA area ranging from 0 to 5.25. Also, to express both the radius and length of the AAA independent of the subject’s nominal arterial size using a single metric, we defined the volumetric severity index (VSI) as:

(2) $$VSI\left(\%\right)=\frac{1}{2.625}\left(\frac{V_{AAA}}{V_{0}}-1\right)\times 100$$

where $V_{AAA}$ is the AAA volume, and $V_{0}$ is the volume of the corresponding artery in the absence of the AAA. The normalizing factor 2.625 corresponds to the maximum possible value of $\left(\frac{V_{AAA}}{V_{0}}-1\right)$ when $l_{AAA}=1$ and $A_{SL}=5.25$. In this way, VSI captures the relative volumetric dilation due to AAA formation, normalized to individual anatomy.

Lastly, to introduce sample-to-sample variability (SSV), each recording of a patient was generated by further perturbing the systemic arterial circulation model parameters: by multiplying their subject-specific values (age-specific nominal multiplied by IIV) by random coefficients drawn from a normal distribution with a mean of 1 and a standard deviation of 0.01.

In summary, each patient was defined by inputting age, IIV, and AAA severity parameters to the systemic arterial circulation model. Then, multiple sets of arterial BP waveforms pertaining to the patient were generated by simulating the systemic arterial circulation model with the specified parameters subject to the SSV. Each waveform was generated by simulating the systemic arterial circulation model until steady state was reached and then taking 2 s duration at a sampling frequency of 128 Hz.

#### 2.1.2. Generation of Brachial and Tibial Pulse Volume Recording Signals

We used the brachial and tibial BP waveform signals (see Section 2.1.1) in conjunction with the brachial and tibial viscoelastic BP-PVR models, developed in a prior work [27], to generate the corresponding brachial and tibial PVR waveform signals (Figure 1). The viscoelastic BP-PVR model simulates the effect of the artery-tissue-cuff interface on the arterial BP waveforms at the PVR measurement sites. The transfer functions governing the viscoelastic BP-PVR relationships at the brachial and tibial sites are given by:

(3) $$Y_{B}\left(s\right)=\frac{E_{B2}+\eta_{B}s}{E_{B1}E_{B2}+\left(E_{B1}+E_{B2}\right)\eta_{B}s}P_{B}\left(s\right),\;Y_{T}\left(s\right)=\frac{1}{E_{T}+\eta_{T}s}P_{T}\left(s\right)$$

where $Y_{B}$ and $Y_{T}$ are brachial and tibial PVR signals, respectively, $P_{B}$ and $P_{T}$ are brachial and tibial BP signals, respectively, $E_{B1}$ and $\eta_{B}$ are the elastic and damping constants of the spring and dashpot connected in parallel in the standard linear solid model, $E_{B2}$ is the elastic constant of the spring connected in series with the dashpot in the standard linear solid model, $E_{T}$ and $\eta_{T}$ are the elastic and damping constants of the spring and dashpot connected in parallel in the Voigt model, and s is the Laplace variable.

We generated synthetic viscoelastic models, replicating the viscoelastic BP-PVR relationships in a patient, by first sampling the five model parameters in Equation (3) from the parameter distributions associated with real patients derived in our prior work (see the Supplementary Material) [28]. Then, to introduce SSV, we modeled each parameter using a lognormal distribution centered at the (sampled) subject-specific value and assigned a coefficient of variation of 0.01. The viscoelastic BP-PVR models parameterized by the random samples drawn from these lognormal distributions were then used to generate brachial and tibial PVR waveform signals.

#### 2.1.3. Data Summary

We generated arterial BP and PVR waveform signals to train, test, and compare two variants of the DL-enabled AAA diagnosis algorithm (one based on brachial and tibial BP waveforms and the other based on brachial and tibial PVR waveforms). First, we created a cohort of 200 patients by randomly assigning each subject an age between 40 and 80 years, which determined the age-dependent nominal values of their systemic arterial circulation model parameters. Second, we applied the IIV coefficients to these nominal parameters to introduce subject-specific variations in heart rate, stroke volume, vascular geometry, terminal loads (i.e., vascular compliances and peripheral resistances), and arterial stiffness. For each subject, we then assigned an AAA severity level by specifying a VSI ranging from 0% to 100%, based on which we randomly selected the corresponding AAA length ($l_{AAA}$) and derived the maximum luminal area increase ($A_{SL}$), and modified the TL_31_ geometry accordingly. We also changed the stiffness of TL_31_ subject to AAA by multiplying its Young’s modulus by a randomly sampled *K_E_*. Third, we sampled the viscoelastic BP-PVR model parameters for brachial and tibial BP-PVR relationships from pre-fitted distributions (see Section 2.1.2) to complete the definition of each patient. To introduce sample-to-sample variability, we generated 100 perturbed realizations per patient by drawing the parameters of the systemic arterial circulation plus BP-PVR model from their respective distributions centered at the subject-specific values with a coefficient of variation of 0.01. Finally, we simulated the systemic arterial circulation plus BP-PVR model 20,000 times (200 patients × 100 samples) to generate 20,000 sets of arterial BP and PVR waveform signals pertaining to all the patients subject to all the SSVs. These arterial BP and PVR waveform signals, along with their associated AAA severity levels and parameters, were used as the labeled training data for the DL-enabled AAA diagnosis algorithm. An additional dataset, generated using the same procedure and of equal size but without AAA severity labels, served as the unlabeled training data (see Section 2.2).

Second, we generated the validation data in the same way as described above. The resulting 20,000 (=200 × 100) arterial BP and PVR waveform signals along with the corresponding AAA severity levels and parameters formed the validation data. We used the validation data to tune the hyperparameters in the DL-enabled AAA diagnosis algorithm.

Third, we generated the test data by sampling 3,000,000 patients, each defined by a randomly assigned age, IIV coefficients, AAA severity level, and viscoelastic BP-PVR model parameters. The resulting 3,000,000 arterial BP and PVR waveform signals along with the corresponding AAA severity levels and parameters formed the test data. The rationale behind generating a large amount of test data was to extensively evaluate the feasibility of DL-enabled pulse wave analysis approach to AAA diagnosis based on arterial BP and PVR waveform signals.

It is noted that we sampled the AAA geometry and stiffness parameters independently, as there is no correlation between AAA diameter and AAA stiffness [12].

### 2.2. Abdominal Aortic Aneurysm Diagnosis Algorithm

To enable AAA diagnosis using non-invasive arterial pulse waveforms, we leveraged the novel continuous property-adversarial regularization (CPAR) method for robust and efficient training of deep neural networks introduced in [23]. The effectiveness of the CPAR method can be found in a prior report [23]. Our AAA diagnosis algorithm examines brachial and tibial PVR waveforms using a convolutional neural network (CNN) trained with the CPAR method.

**Figure 2:**
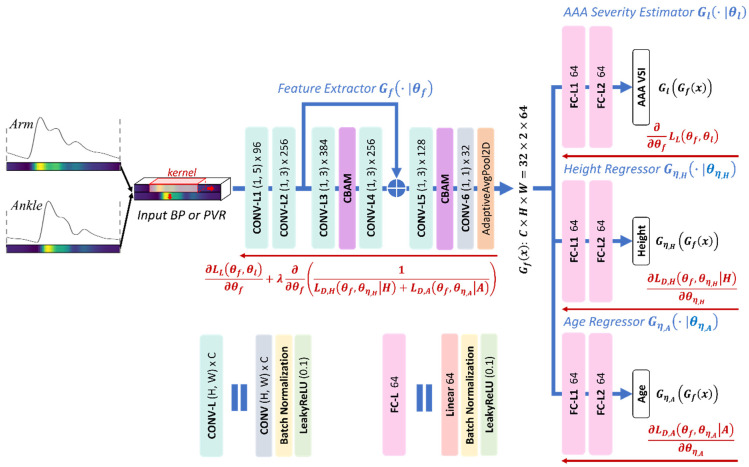
Convolutional neural network (CNN) architecture and its training based on the continuous property-adversarial regularization (CPAR) method, described in Equations (4) and (5). The network processes vertically concatenated brachial and tibial arterial blood pressure (BP) or pulse volume recording (PVR) waveforms through five convolutional layers with a skip connection, two attention modules (CBAM), and a compression phase, yielding latent features of size (32 × 2 × 64). These features are passed to three regressor heads: one for abdominal aortic aneurysm (AAA) volumetric severity index (VSI) estimation and two adversarial regressors for continuous property-adversarial regularization, enforcing invariance to subject height and age. Blue and red arrows show the flow of the input data and gradients in training, respectively.

The CNN architecture is essentially a one-dimensional adaptation of AlexNet [29], which includes 5 convolutional layers for feature extraction and 3 fully connected layers for label prediction (Figure 2). The inputs to the CNN are brachial and tibial PVR waveforms concatenated vertically, forming a tensor of shape (channel = 1, height = 2, width = 256). For feature extraction, we used five convolutional layers with a kernel size of 1 × 5 initially and thereafter 1 × 3, using a stride of 1, a dilation of 3, and a padding to preserve temporal length. The one-dimensional kernels move along each row of the input tensor and preserve its two-row layout through the feature extraction layer, leading to independently extracted features from the brachial and tibial sites. We further improved training stability and feature representation by adding a skip connection, an attention module, and a compression phase to the convolutional layers. First, a skip connection was added between the second and fourth layers to promote faster convergence and smoother training. Second, a Convolutional Block Attention Module (CBAM), introduced in [30], with 7 × 7 kernels and a reduction factor of 16, was added after the third and fifth layers to enable attention-based feature refinement both temporally and channel-wise. Lastly, the output of the last convolution layer is downsampled using a 1 × 1 convolution followed by average pooling to reduce the number of parameters in the subsequent fully connected layers. The resulting latent features, a tensor of shape (channel = 32, height = 2, width = 64), are then flattened and passed to three parallel regressor heads. Each regressor consists of three fully connected layers with 64, 64, and 1 neuron(s), respectively. One of the heads is used to estimate the AAA VSI, while the other two heads serve as adversarial components for regularization furnished by the CPAR method (Figure 2). During training, the two adversarial regressors are trained to predict the subject’s height and age, while the feature extractor is trained to make these regressors fail. This is implemented through our choice of cost functions and update laws in Equations (4) and (5). In effect, the feature extractor learns to generate representations that are useful for predicting AAA VSI but uninformative about height and age. Once training is completed, the adversarial heads are turned off, leaving only the AAA VSI regressor active during inference. Thus, the CPAR framework acts solely as a regularizer that enforces invariance against confounders during training without adding any computational overhead during testing.

The feature extraction layers contain approximately 810,000 parameters, while each of the three regressor heads has around 270,000 parameters. To mitigate the risk of overfitting, we employed early stopping, batch normalization, and the CPAR method. The CPAR method extends the notion of domain-adversarial training framework originally introduced in [31], which promotes domain-invariant feature representations by adversarially minimizing the CNN’s ability to infer domain labels from latent features. In contrast to the original method that operates only over discrete domains, the CPAR method generalizes the principle to continuous-valued variables by reformulating the domain classification task as a continuous property regression problem [23]. The CPAR method and similar approaches have shown advantages in synthetic and real-world medical datasets [23,32]. In this work, we regularized the feature extractor to be invariant to height and age, both of which are known to affect arterial length and wave propagation, thereby altering arterial BP and PVR waveform morphology [33,34]. We used labeled data to jointly optimize the AAA VSI regression head and the feature extractor, while we used both labeled and unlabeled data to perform adversarial regularization. Consequently, the two adversarial regressors were trained on both labeled and unlabeled data, whereas the AAA VSI regressor was trained exclusively on the labeled data. In this way, the CPAR method allows us to harness additional unlabeled data, which are more widely available as they require only the PVR recordings and rudimentary demographic attributes such as age and height, to impose stronger regularization without costly measurements. To implement the CAPR method, we formulated the training process as a joint optimization problem involving a label prediction loss and two disturbance regression losses corresponding to subject height and age (Figure 2):

(4) $$\begin{array}{c} L_{L}\left(\theta_{f},\theta_{l}\right)=\frac{1}{N}\sum_{i=1}^{N}{\left(y_{i}-G_{l}\left(G_{f}\left(x_{i}\right)\right)\right)}^{2}\\ L_{D,H}\left(\theta_{f},\;\theta_{\eta ,H}|H\right)=\frac{1}{N}\sum_{i=1}^{N}\log\left(\frac{1}{1-tanh\left|H_{i}-G_{\eta ,H}\left(G_{f}\left(x_{i}\right)\right)\right|}\right)\\ L_{D,A}\left(\theta_{f},\;\theta_{\eta ,A}|A\right)=\frac{1}{N}\sum_{i=1}^{N}\log\left(\frac{1}{1-tanh\left|A_{i}-G_{\eta ,A}\left(G_{f}\left(x_{i}\right)\right)\right|}\right) \end{array}$$

where $L_{L}\left(\theta_{f},\theta_{l}\right)$, $L_{D,H}\left(\theta_{f},\;\theta_{\eta ,H}|H\right)$, and $L_{D,A}\left(\theta_{f},\;\theta_{\eta ,A}|A\right)$ are the loss functions pertaining to the label predictor and the disturbance domain regressors associated with height (H) and age (A), respectively; $G_{l}\left(\cdot \right)$, $G_{\eta ,H}\left(\cdot \right)$, $G_{\eta ,A}\left(\cdot \right)$, and $G_{f}\left(\cdot \right)$ are the mappings from latent features to the label, height (H), and age (A), and from input to latent features, respectively; $\theta_{l}$, $\theta_{\eta ,H}$, $\theta_{\eta ,A}$, and $\theta_{f}$ are the trainable parameters in the label prediction layer, disturbance regressors for height and age, and feature extraction layer, respectively. Correspondingly, each set of parameters is updated according to:

(5) $$\begin{array}{c} \theta_{l}^{*}=\arg\underset{\theta_{l}}{\min}\;L_{L}\left(\theta_{f},\theta_{l}\right)\\ \theta_{\eta ,H}^{*}=\arg\underset{\theta_{\eta ,H}}{\min}\;L_{D,H}\left(\theta_{f},\;\theta_{\eta ,H}|H\right)\\ \theta_{\eta ,A}^{*}=\arg\underset{\theta_{\eta ,A}}{\min}\;L_{D,A}\left(\theta_{f},\;\theta_{\eta ,A}|A\right)\\ \theta_{f}^{*}=\arg\underset{\theta_{f}}{\min}\;L_{L}\left(\theta_{f},\theta_{l}\right)+\lambda \frac{1}{L_{D,H}\left(\theta_{f},\;\theta_{\eta ,H}|H\right)+L_{D,A}\left(\theta_{f},\;\theta_{\eta ,A}|A\right)} \end{array}$$

where λ is the regularization weight that modulates the strength of the CPAR method relative to label prediction.

We trained the CNN using the ADAM optimizer and the training and validation data described in Section 2.1. Hyperparameter tuning was performed once using Optuna [35], a framework for automated optimization, to select the optimal values for the ADAM optimizer parameters and the CPAR regularization weight λ in Equations (4) and (5). The selected hyperparameters, shown in Table 1, were then fixed and applied uniformly across all subsequent training runs. To assess robustness, we repeated the data generation and training procedures 10 times, resulting in 10 independently trained CNN models. Training was performed using approximately one-seventh of an NVIDIA A100 GPU with 5 GB of memory. Each model required 30 to 60 min to train, depending on early-stopping termination based on validation loss (patience = 25 epochs, max allowable epochs = 100). The final evaluation of AAA diagnosis performance was based on the models’ predictions on the test data. To compare the efficacy of PVR-based AAA diagnosis with arterial BP-based AAA diagnosis, we repeated the above procedure using brachial and tibial BP waveform signals.

### 2.3. Data Analysis

To investigate the plausibility of the age dependence of the arterial anatomical, biomechanical, and hemodynamic parameters in the systemic arterial circulation model, we simulated the systemic arterial circulation model for each age value between 30 and 80 years (in 5-year increments) 5^8^ times by sampling five equally spaced values of the eight IIV coefficients. Then, we calculated the descriptive statistics of aortic systolic and pulse pressures, aortic–brachial pulse pressure amplification, and carotid–femoral PWV pertaining to each age group and compared them with the in vivo ranges reported in a prior work [25].

**Figure 3:**
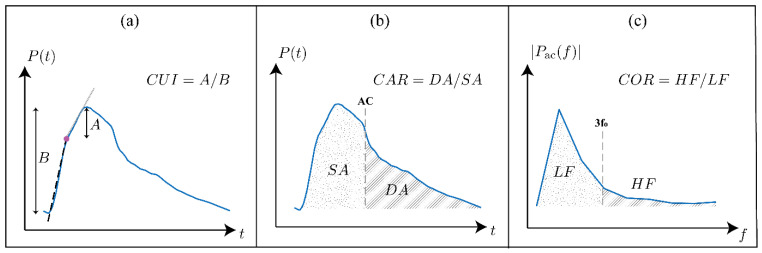
Illustration of the three waveform-derived features evaluated in this study. (**a**) Carotid upstroke index (CUI): the foot-to-peak interval of the carotid arterial blood pressure (BP) waveform is divided at the optimized intersection point (filled dot), least-squares lines are fitted to the two upstroke regions, and CUI is calculated as the ratio of the peak-to-intersection amplitude to the pulse pressure. (**b**) Carotid area ratio (CAR): the systolic (dotted) and diastolic (hatched) areas under the carotid arterial BP waveform are calculated, and CAR is calculated as the diastolic area divided by the systolic area. (**c**) Carotid oscillatory ratio (COR): the carotid arterial BP waveform is transformed into the frequency domain after mean subtraction, and COR is calculated as the ratio of spectral energy in the high-frequency band (defined as 3–8 times the fundamental frequency = HR/60) to that in the low-frequency band (defined as 0–3 times the fundamental frequency).

To investigate the validity of the arterial systemic circulation model in the context of AAA, we analyzed the shape of arterial BP waveforms in our generated data. Prior studies have shown that AAA alters the morphology of the arterial BP waveforms [13,36,37]. These changes are mainly due to the increase in the diameter of the infra-renal aorta, which is also seen as increased compliance and reduced stiffness of the affected region. The decreased impedance of the AAA region alters the overall BP propagation dynamics: specifically, the compliant AAA region further acts as a partial reflection site for the passing BP waves due to the substantial local impedance mismatch. We quantitatively studied the change in the propagation and reflection characteristics of the arterial system caused by the presence of AAA by extracting four features from the arterial BP waveforms previously shown or hypothesized to correlate with the diameter of the infra-renal aorta. Three of these features, carotid–femoral PWV [14,38,39], carotid upstroke index (CUI) [37], and carotid area ratio (CAR) [37] are time-domain metrics studied in prior studies (Figure 3), while carotid oscillatory ratio (COR) is a frequency-domain feature proposed in this work.

1. Carotid–femoral PWV was calculated using the intersecting tangent method [40] applied to carotid and femoral arterial BP waveforms. PWV is a well-established surrogate for aortic stiffness and has been shown to decrease in the presence of AAA [14,38,39].
2. CUI was computed by dividing the foot-to-peak upstroke of the carotid arterial BP waveform into two regions and then fitting a line to each region (Figure 3a). The segmentation point was selected as the time point that minimizes the total root-mean-squared error of both linear fits. CUI was then calculated as the ratio of the peak-to-intersection amplitude to the pulse pressure and should generally increase with the presence of AAA, capturing the change in the early systole period of the carotid arterial BP waveform caused by the negative wave reflection due to the presence of AAA [37].
3. CAR was calculated as the ratio of the area under the carotid arterial BP waveform between diastole and systole (Figure 3b). CAR should generally increase with increasing AAA severity as the negative reflected wave arrives at the upstream measurement site around early systole [37].
4. To calculate COR, the carotid arterial BP waveform was mean-subtracted and transformed into the frequency domain. Then, COR was calculated as the ratio of the spectral energy in the high-frequency band (defined as 3–8 times the fundamental frequency = HR/60) to that in the low-frequency band (defined as 0–3 times the fundamental frequency) (Figure 3c). COR is expected to increase with AAA severity because of the presence of more oscillations in the arterial BP waveforms due to increased wave reflections [13].

We evaluated the performance of our DL-enabled algorithm for AAA diagnosis based on PVR waveform analysis (DL-PVR) to (i) assess AAA severity and (ii) detect AAA. We reported the results as the aggregated (i.e., averaged) performance of 10 independently trained CNNs on the test data described in Section 2.1.3. We measured the performance of AAA severity assessment in terms of (i) the Pearson correlation coefficient (*ρ*), (ii) the mean absolute error (MAE), and (iii) the root mean squared error (RMSE) between predicted vs. reference VSI values. We measured the performance of AAA detection in terms of sensitivity, specificity, accuracy, positive predictive value (PPV), negative predictive value (NPV), F1 score, and the area under the receiver operating characteristic (ROC) curve and the precision–recall curve (PRC). To examine the robustness of detection performance across varying labeling thresholds, we tested AAA labeling cutoffs at 20%, 30%, 40%, 50%, 60%, 70%, and 80% VSI levels. At each labeling threshold, we redefined the binary classification labels by designating subjects with AAA VSI exceeding the given labeling threshold as positive cases and those below the given labeling threshold as negative cases. Then, we recalculated all corresponding classification metrics accordingly. To compare it with a DL-enabled algorithm for AAA diagnosis based on arterial BP waveform analysis (DL-ABP) (which may exhibit superior performance to its PVR counterpart), we calculated the above-mentioned metrics for the CNNs trained using brachial and tibial BP waveforms (see Section 2.2) and compared them with those for the CNNs trained using brachial and tibial PVR waveforms.

We performed the feature analysis using carotid and femoral arterial BP waveforms, as prior work has studied these sites to reveal the influence of AAA on them. In contrast, we conducted the DL analysis using brachial and tibial PVR waveforms, as these sites provide more practical options for cuff-based acquisition in point-of-care and home settings.

## 3. Results

**Figure 4:**
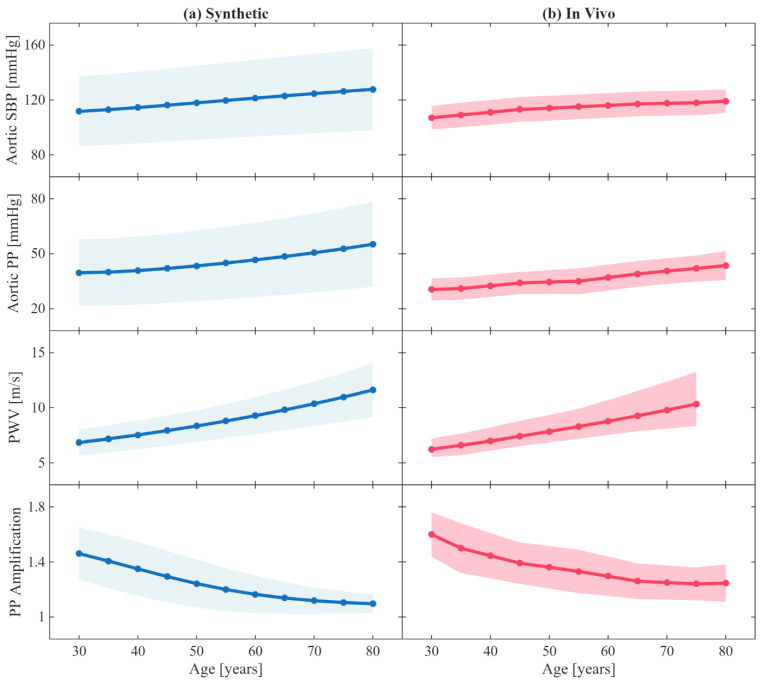
Age-related changes in key hemodynamic parameters. (**a**) Synthetic results from the age-adjusted arterial circulation model, where arterial geometry (length and radius), stiffness, peripheral resistance and compliance, and stroke volume were made age-dependent using the empirical equations reported in a prior work [25]. (**b**) Corresponding age-related trends from in vivo measurements reported in a prior work [25]. Solid lines represent mean values across patients, while the filled areas indicate ±1 standard deviation. SBP: systolic blood pressure. PP: pulse pressure. PWV: carotid–femoral pulse wave velocity. PP amplification is measured between aorta and brachial artery.

**Figure 5:**
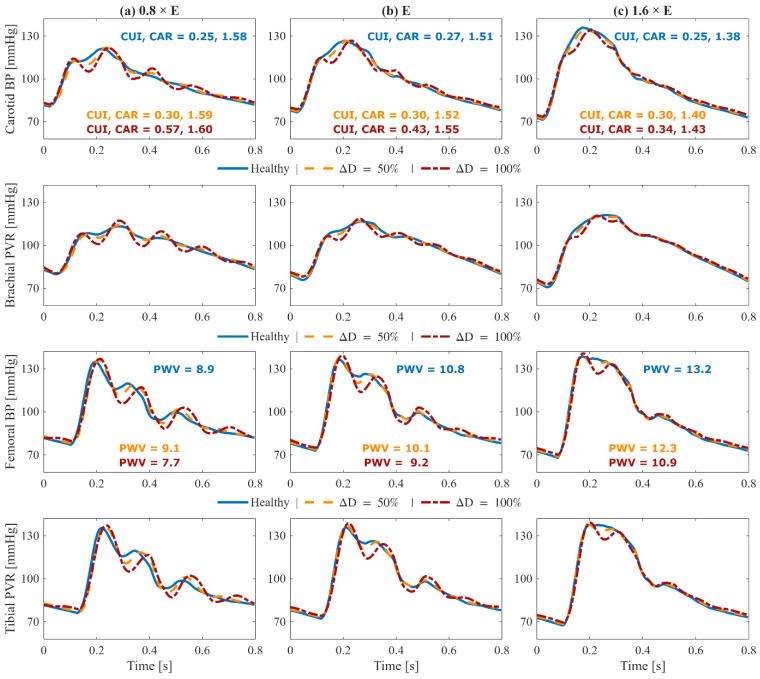
Effect of abdominal aortic aneurysm (AAA) on simulated pulse volume recording (PVR) and arterial blood pressure (BP) waveforms pertaining to a 70-year-old subject with nominal physiological parameters: (1st row) carotid arterial BP, (2nd row) brachial PVR, (3rd row) femoral arterial BP, and (4th row) tibial PVR. Each column corresponds to a different arterial tree stiffness (E): reduced stiffness ((**a**), 0.8 × E), nominal stiffness ((**b**), E), and elevated stiffness ((**c**), 1.6 × E). In each plot, three waveforms are shown: healthy (solid blue), 50% expansion in maximum TL_31_ diameter (dashed orange), and 100% expansion in maximum TL_31_ diameter (dash-dotted red). Key waveform features are annotated in carotid and femoral arterial BP panels: carotid upstroke index (CUI), carotid area ratio (CAR), and carotid–femoral pulse wave velocity (PWV). The results demonstrate how AAA changes upstream and downstream arterial BP and PVR waveforms.

**Figure 6:**
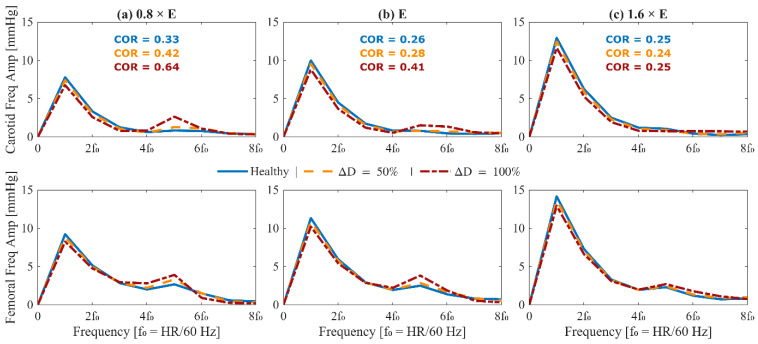
Frequency-domain analysis of arterial blood pressure waveforms in the presence of abdominal aortic aneurysm (AAA) for a 70-year-old subject with nominal physiological parameters. (top) Amplitude spectra of mean-subtracted carotid arterial blood pressure. (bottom) Amplitude spectra of mean-subtracted femoral arterial blood pressure. Each column corresponds to a different arterial stiffness: reduced stiffness ((**a**), 0.8 × E), nominal stiffness ((**b**), E), and elevated stiffness ((**c**), 1.6 × E). In each plot, three waveforms are shown: healthy (solid blue), 50% expansion in maximum TL_31_ diameter (dashed orange), and 100% expansion in maximum TL_31_ diameter (dash-dotted red). The carotid oscillatory ratio (COR) is annotated in the upper row. Frequency is normalized to the heart rate fundamental (f_0_ = HR/60 Hz). The results demonstrate how AAA alters harmonic content and energy distribution in upstream and downstream arterial blood pressure waveforms.

**Figure 7:**
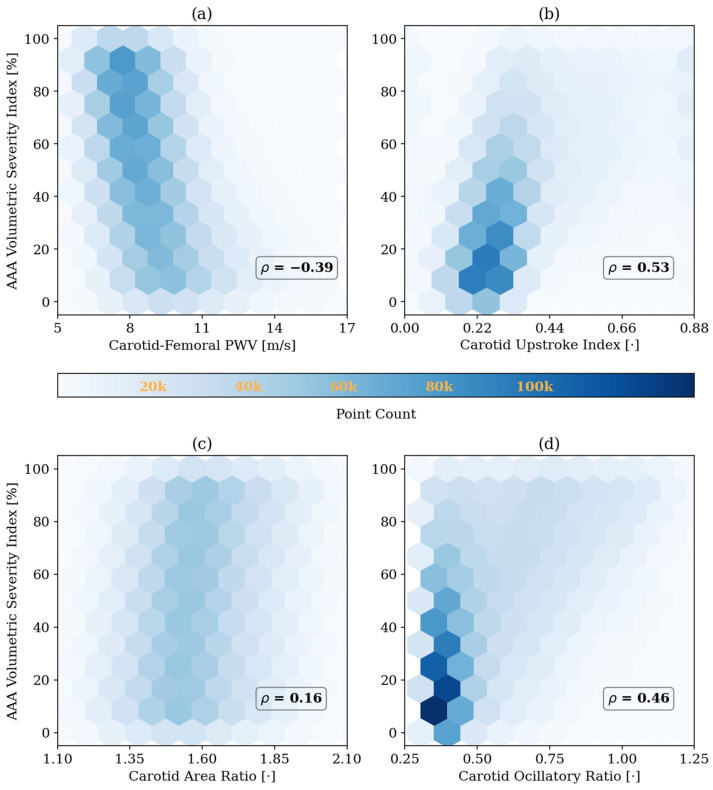
Correlation between abdominal aortic aneurysm (AAA) volumetric severity index and four representative arterial BP waveform features in the test data: (**a**) carotid–femoral pulse wave velocity (PWV), (**b**) carotid upstroke index, (**c**) carotid area ratio, and (**d**) carotid oscillatory ratio. Pearson correlation coefficients (*ρ*) are shown in each panel. The results highlight how morphological features in the arterial blood pressure waveforms are influenced by AAA severity. All reported correlations are statistically significant (*p* < 0.05).

**Figure 8:**
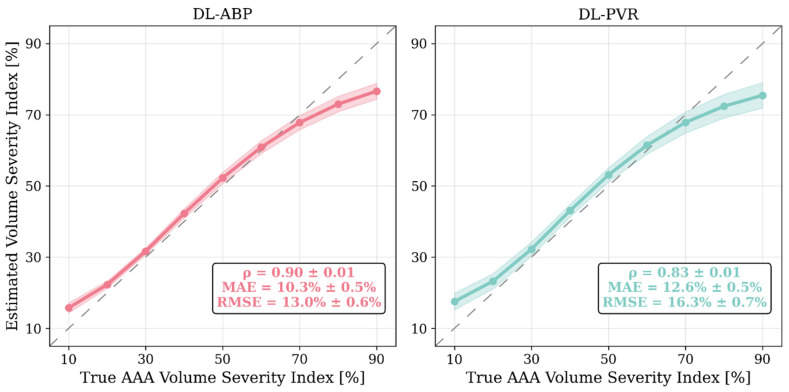
Comparison of abdominal aortic aneurysm (AAA) severity estimation performance of the deep learning-enabled AAA diagnosis algorithm using arterial blood pressure waveforms (DL-ABP) versus pulse volume recording waveforms (DL-PVR). Solid lines show the mean predicted value across the 10 independently trained CNNs for each true-severity bin (5–15%, 15–25%, …, 85–95%), shaded areas indicate their ±1 standard deviation, and the dashed diagonals denote the ideal response (y = x). Pearson correlation coefficient (*ρ*), mean absolute error (MAE), and root-mean-squared error (RMSE) are computed per CNN on the test data and then summarized as mean ± standard deviation (MAE and RMSE reported in %VSI).

**Figure 9:**
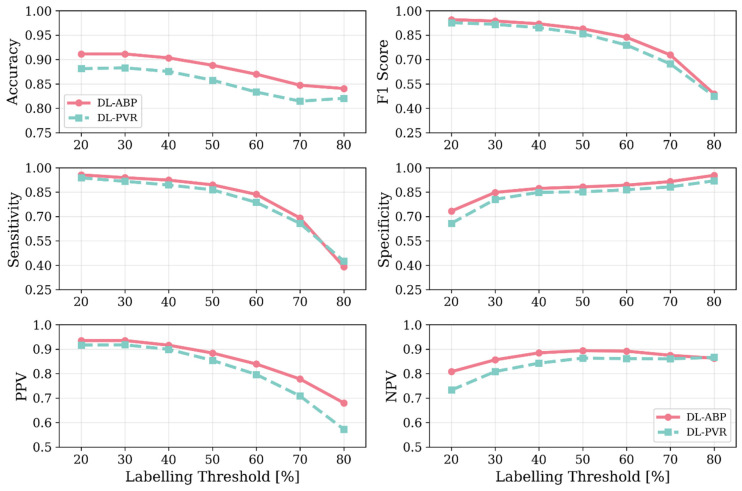
Accuracy, F1 score, sensitivity, specificity, positive predictive value (PPV), and negative predictive value (NPV) values associated with DL-enabled arterial BP waveform analysis (DL-ABP) vs. DL-enabled PVR waveform analysis (DL-PVR) at 20–80% AAA severity levels as the labeling threshold. The metrics were computed with the classifier threshold set equal to the labeling threshold. Note that the labeling threshold controls how positive and negative classes are defined in the dataset (a case is labeled positive if VSI exceeds the labeling threshold and negative otherwise).

**Figure 10:**
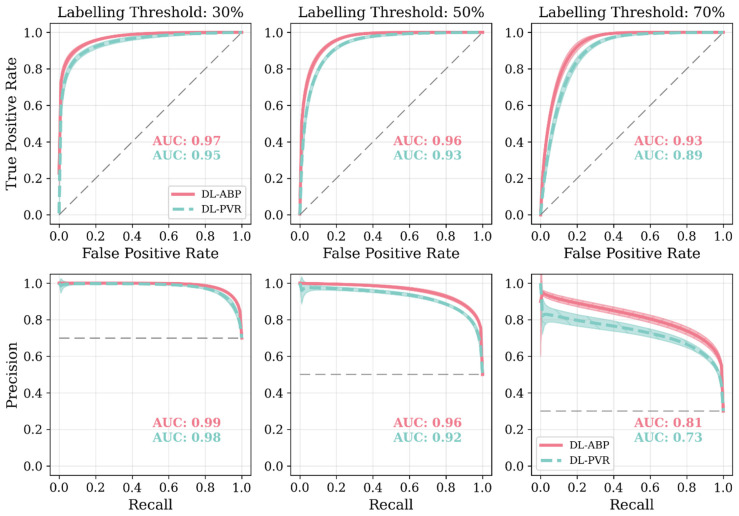
Receiver operating characteristic plots (ROCs) and precision–recall curves (PRCs) associated with DL-enabled arterial BP waveform analysis (DL-ABP) vs. DL-enabled PVR waveform analysis (DL-PVR) at 30%, 50%, and 70% AAA severity levels as the labeling threshold (i.e., the VSI thresholds used to define positive versus negative cases). The area under the curve (AUC) value is annotated in each plot. Solid lines represent the average, while shaded areas represent the standard deviation across 10 tests. The gray dashed line in the ROC plots represents a classifier with no discriminative ability, and in the PRC plots it marks the baseline precision expected from random guessing (equal to the prevalence of positives). Note that the labeling threshold sets the ground truth (a case is labeled positive if VSI exceeds the labeling threshold and negative otherwise). Once this ground truth is fixed, we compute ROC and PRC by sweeping the classifier’s decision threshold over the algorithm’s predicted VSI values and, at each decision threshold, we record both true positive rate and false positive rate for ROC and both precision and recall for PRC. In other words, the labeling threshold controls how positive and negative classes are defined in the dataset, whereas the ROC and PRC curves show how performance changes as we vary the classifier’s decision rule over those fixed labels.

Figure 4 shows the change in key hemodynamic parameters and their variability vs. age within our synthetic data compared to in vivo data reported in the literature. Figure 5 shows representative examples of BP waveforms at carotid and femoral arteries and PVR waveforms at brachial and tibial arteries pertaining to 0%, 50%, and 100% increase in maximum TL_31_ diameter with respect to different values of arterial stiffness. Figure 6 shows representative examples of the frequency amplitude of BP waveforms at carotid and femoral arteries pertaining to 0%, 50%, and 100% increase in maximum TL_31_ diameter with respect to different values of arterial stiffness. Figure 7 shows the contour plots between four representative arterial BP waveform features and the AAA VSI in the test data. Figure 8 and Table 2 compare the performance metrics of DL-ABP vs. DL-PVR pertaining to AAA severity estimation and AAA detection, respectively. Table 3 compares the Pearson correlation coefficient between four arterial BP waveform features and two AAA severity metrics (namely, maximum diameter and VSI). Figure 9 shows sensitivity, specificity, accuracy, PPV, NPV, and F1 score values associated with DL-PVR and DL-ABP at 20–80% AAA severity levels as the labeling threshold. Figure 10 shows ROC and PRC associated DL-PVR and DL-ABP at 30%, 50%, and 70% AAA severity levels as the labeling threshold.

## 4. Discussion

Abdominal aortic aneurysm (AAA) is a silent yet life-threatening dilation of the infrarenal aorta whose rupture carries 85% mortality and ranks AAA among the ten leading causes of death in men older than 55 years in the US [4]. Definitive diagnosis and surveillance hinge upon imaging modalities that are accurate but capital-intensive, operator-dependent, and therefore chronically under-utilized; around 60% of privately insured patients in the US do not receive the recommended AAA screening, especially those living in non-metropolitan areas [9]. The resulting diagnostic gap underscores an urgent need for inexpensive, operator-agnostic tools that can flag high-risk individuals early enough to justify confirmatory imaging. Physiological waveform analysis may offer such a path because an AAA modifies aortic geometry and compliance, leaving measurable imprints on upstream and downstream BP and blood flow waves. Recent studies have begun to leverage these signals, yet most approaches still rely on multiple invasive measurements [15,16], which remain costly and impractical, or they use PPG waveforms that are highly susceptible to noise and peripheral vasoconstriction and may lack sufficient information to detect subtle aneurysm-induced changes [17,18]. Hence, there is a need to enable AAA diagnosis using affordable, convenient, and non-invasive arterial pulse waveform signals. Hence, we investigated the feasibility of AAA diagnosis based on the analysis of non-invasive arterial pulse waveforms called PVR signals, which can be easily measured using low-cost BP cuff devices.

### 4.1. Mathematical Model of Arterial Circulation: Physiological Plausibility

To evaluate the plausibility of the role of age within our systemic arterial circulation model, we examined the age-related trends in hemodynamic characteristics of our synthetic arterial BP waveforms in comparison with the in vivo trends reported in the literature (Figure 4). Consistent with prior in vivo and in silico observations [25], carotid–femoral PWV, aortic systolic pressure, and aortic pulse pressure increased progressively with age, reflecting arterial stiffening, while pulse pressure amplification between the aorta and brachial artery decreased with age, reflecting the increase in late systolic aortic pressure caused by elevated PWV. The range of synthetic aortic systolic and pulse pressures was larger than the in vivo range, which is not ideal but may at least allow us to conservatively encompass large enough aortic systolic and pulse pressure range in the development and evaluation of our AAA diagnosis algorithm. The adequate agreement between our synthetic results and the in vivo trends in the literature supports the physiological plausibility of our systemic arterial circulation model in capturing age-dependent arterial hemodynamics and highlight the relevance of the demographics in our data, focusing on older adults (40–80 years old) as the primary population susceptible to AAA.

To ensure that the simulated arterial BP waveforms in the presence of AAA are physiologically plausible, we first verified whether our mathematical model reproduces the hemodynamic signatures that AAA imparts on arterial BP waveforms. These signatures are caused by AAA-induced alterations in wave propagation and reflection characteristics in the aorta [13,37,39]. In particular, a local expansion in the abdominal aorta increases compliance, producing a segment of low characteristic impedance. As BP waves enter this compliant segment, they meet a sudden drop in impedance, and as they exit, they face a return to higher impedance; both boundaries act as reflection sites that send part of the BP wave back toward the upstream aorta [11,13]. The elevated compliance leads to a decrease in PWV [14,38,39], while the wave reflections lead to visually recognizable morphological changes including the presence of more oscillations, strong inflection points, and decreased systolic area under the curve in the BP waveforms [13,37]. These qualitative observations were measured through a set of representative features to verify the plausibility of our mathematical model. A total of 4 features pertaining to arterial BP waveforms were investigated, including PWV, CUI, CAR, and COR (see Section 2.3). In Figure 5 and Figure 6, these features are shown alongside their corresponding carotid and femoral arterial BP waveforms at three AAA severity levels and three arterial tree stiffness levels. Figure 5 also shows brachial and tibial PVR signals, which are influenced by AAA in a similar fashion to arterial BP waveforms. Consistent with theory [13,37,38,39], PWV decreased while CUI, CAR, and COR increased with AAA severity at all arterial stiffness levels. Expectably, these features were modulated through arterial stiffness as well; within these sample cases, PWV was influenced by both AAA severity and arterial stiffness to a relatively similar extent, CUI was only fairly affected by arterial stiffness and more by AAA severity, CAR was modulated more by arterial stiffness than by AAA severity, while COR was only changing with AAA severity in normal to lower arterial stiffness levels. The correlation of these features with AAA severity was evaluated beyond the illustrative cases; in our extensive test data, AAA severity showed: (i) a fair negative correlation with PWV (ρ=−0.39), (ii) a moderate positive correlation with CUI (ρ=0.53), (iii) a weak positive correlation with CAR (ρ=0.16), and (iv) a moderate positive correlation with COR (ρ=0.46) (Figure 7). Notably, the relationship between AAA severity vs. PWV and CUI was relatively linear; by contrast, CAR showed almost no linear trend, while COR exhibited a complex spread. COR’s dense concentration in the [0.25–0.5] range remained unaffected by the increases in VSI up to 60%, which is consistent with the annotated values in the illustrated normal and elevated stiffness levels (Figure 6b,c). Taken together, the observed patterns mostly align with the established pathophysiological observations in the literature (at least qualitatively), while also exhibiting substantial variability, and overall support the plausibility of the synthetic arterial pulse waveforms that served as the basis for the subsequent deep learning analyses.

### 4.2. Efficacy of DL-Enabled PVR/BP Waveform Analysis-Based AAA Diagnosis

Our results based on extensive and physiologically diverse in silico test data demonstrate that AAA may be adequately detected by analyzing PVR waveforms at extremity sites. In particular, the DL-PVR algorithm provided a continuous estimate of aneurysm severity that was closely aligned with the ground truth, with a Pearson correlation coefficient of *ρ* = 0.83 ± 0.01 and a mean absolute error of 12.6 % ± 0.5 % (Figure 8). Beyond regression, the algorithm also demonstrated adequate detection performance. At 30%, 50%, and 70% VSI thresholds (which corresponds to 80%, 130%, and 180% increase in abdominal aortic volume relative to its healthy counterpart), accuracy remained relatively stable across the evaluated thresholds (>0.80). However, sensitivity, PPV, specificity, and NPV exhibited a non-ideal trade-off, with sensitivity and PPV decreasing while specificity and NPV increasing as the labeling threshold increased (Figure 9). The decrease in PPV can be at least partly attributed to the decreased prevalence of the positive class at higher labeling thresholds, whereas the decrease in sensitivity reflects the DL-PVR’s tendency to underestimate the severity in cases with a VSI close to 1 (Figure 8), causing true positives to be missed under stricter detection criteria. Consequently, the F1 score degraded with increasing threshold, capturing the combined effects of reduced sensitivity and PPV. Nevertheless, F1 score, PPV, and NPV values above 0.80 at 30–50% VSI thresholds are still promising, making the early identification of at-AAA-risk individuals possible. Notably, 30% VSI may be an adequate practical threshold that justifies referral for confirmatory ultrasound imaging (pending prospective clinical validation) as it corresponds to a 60% relative increase in the maximum diameter of the infrarenal aorta (assuming the AAA profile in Equation (1) and $l_{AAA}=1$). As shown in Figure 10, at 30% VSI labeling threshold, our DL-enabled algorithm is associated with a ROC profile superior to manual palpation (which is a well-known physical examination method for AAA detection accounting for about one third of new diagnoses): at 75% specificity level, our DL-enabled algorithm is associated with 93% sensitivity, which is much higher than manual palpation (68%) [41]. Additionally, the DL-PVR algorithm achieved ROC-AUC values of 0.95, 0.93, and 0.89, with the corresponding PRC-AUCs of 0.98, 0.92, and 0.73 at 30%, 50%, and 70% VSI labeling thresholds (Figure 10).

When compared with the DL-ABP algorithm trained on the brachial and tibial BP waveforms, DL-PVR exhibited a modest decline in performance. Although the Pearson correlation coefficient decreased from 0.90 to 0.83, and the MAE increased from 10.3% to 12.6%, the algorithm built with PVR still retained strong performance (Figure 8). At 30–50% VSI thresholds, the DL-PVR algorithm resulted in NPV, PPV, and F1 score which were very close to (<5% lower than) those pertaining to their DL-ABP counterparts (Figure 9). The areas under ROC and PRC also dropped only marginally (Figure 10). Overall, such degradation could be attributed to the viscoelastic damping in the arterial vessel wall and the overlying tissues, because: (i) they alter the BP waveform before it reaches the cuff, thereby blunting the morphological cues that the DL algorithm relies on; and (ii) the IIV associated with the viscoelastic damping varies how BP waveforms are altered in different individuals.

### 4.3. Relevance of Volumetric vs. Diameter-Based AAA Severity Metrics to BP Waveform Morphology

Several anatomical metrics can quantify AAA severity. In contrast to the current clinical standards for assessing AAA severity that rely on the maximum AAA diameter and its rate of expansion [1], we adopted a volumetric AAA severity (namely, VSI; Equation (2)) as a metric of AAA severity. The VSI normalizes the aneurysm volume by its neck area and incorporates the lesion’s longitudinal extent. Unlike maximum AAA diameter, the VSI is independent of the patient’s baseline aortic size and may better reflect the overall burden of dilation. To determine which severity metric aligns more closely with arterial BP waveform morphology, we computed the correlations between these severity indices vs. the morphological features representative of AAA (see Section 2.3). Across the four representative waveform features (namely, PWV, CUI, CAR, and COR), the VSI consistently showed stronger correlations (Table 3). We further compared the regression performance of the AAA diagnosis algorithms when developed on the PVR waveforms to estimate the two severity metrics and found that the prediction of the VSI yielded a lower mean absolute error (12.3% vs. 14.5%) and higher Pearson correlation (*ρ* = 0.83 vs. 0.78) compared to maximum AAA diameter. These results suggest that the volumetric AAA severity index may be more tightly coupled to the arterial BP waveform features and offer a more physiologically grounded target for machine learning-based assessment of AAA severity.

### 4.4. Study Limitations

This study was conducted entirely with synthetic data generated by a physiologically grounded but simplified 1-dimensional mathematical model of the arterial tree. While the ability of such a mathematical model to capture the main features of arterial blood pressure and flow waves in the presence of AAA and peripheral artery disease has been previously confirmed against in vitro/in vivo data [13,42], they may not fully capture the physiological heterogeneity observed in real AAAs. As such, the waveform signals may only provide a plausible but not perfectly realistic representation of patient physiology.

The representation of AAA severity in this work was based on an idealized geometry. In reality, AAAs can exhibit asymmetric and tortuous shapes and may also contain intraluminal thrombosis and wall classifications [26]. These features are known to influence biomechanics and rupture risk [43,44,45], and the absence of such features in our work may limit direct translation of the findings to clinical practice.

Our DL-enabled algorithm was trained and tested exclusively on data derived from the same modeling framework. Although we used an extensively large test dataset compared to the training data, there is still a possibility that the reported diagnostic performance metrics are optimistic. In practice, recordings from different patients are subject to comorbidities (e.g., smoking, diabetes, hypertension), sex differences, motion artifacts, noise, and device variability, which were not explicitly represented in the synthetic data in our work, which motivates the prospective validation of our DL-enabled algorithm in real patient cohorts.

To address the above limitations, prospective validation in real patient cohorts will be essential, which will help establish robustness and clinical utility and to determine whether the benefits of the proposed framework hold in practical screening and monitoring scenarios. Currently, we are not aware of any publicly available high-quality and large-scale clinical dataset ideally suited to validate our DL-enabled algorithm. Hence, prospective validation will likely require a large-scale clinical dataset including the measurements of arm and ankle PVR signals as well as the corresponding VSI values collected from AAA patients and matched control subjects. Commercially available arm-ankle cuff devices [46] and advanced signal processing techniques [47] may be employed together to record the PVR signals, while imaging and subsequent analysis may be required to measure VSI. Our results suggest that a dataset collected from 170 AAA patients and the same number of matched control subjects may be adequate for training our DL-enabled algorithm. We anticipate that such a large dataset may need to be collected via multi-hospital collaborations. If the prospective clinical validation is successful, a cuff device capable of automatically measuring and analyzing the PVR signals at both arm and ankle locations may be commercialized and deployed to primary care practices for pre-ultrasound screening and post-surgical monitoring.

## 5. Conclusions

We developed and evaluated a DL-enabled algorithm for AAA diagnosis using arterial BP and PVR waveform signals. We showed the plausibility of the synthetic arterial BP and PVR waveform signals with respect to age and in the presence of AAA using the findings reported in the literature. The DL-enabled algorithm showed promise in detecting and estimating the severity of AAA using brachial and tibial PVR signals easily measurable with existing cuff devices. Future work must investigate the validation of the approach using more realistic data (e.g., real clinical data) and the improvement of the robustness of the approach against confounding factors.

## Figures and Tables

**Table 1:** Selected hyperparameters for the deep learning-enabled AAA diagnosis algorithms using arterial blood pressure (DL-ABP) and pulse volume recording (DL-PVR) waveforms. *β*_1_ and *β*_2_ are the Adam optimizer momentum coefficients. *λ* is the CPAR regularization weight in Equations (4) and (5). $\alpha_{f}$, $\alpha_{l}$, and $\alpha_{\eta }$ are the learning rates for the feature extractor, VSI regressor head, and adversarial regressors, respectively.

	Batch Size	$\beta_{1}$	$\beta_{2}$	λ	$\alpha_{f}$	$\alpha_{l}$	$\alpha_{\eta }$
DL-ABP	16	0.989	0.970	$7.3\times 10^{-4}$	$2.2\times 10^{-4}$	$3.6\times 10^{-5}$	$5.5\times 10^{-6}$
DL-PVR	16	0.950	0.950	$6.5\times 10^{-4}$	$1.2\times 10^{-3}$	$1.1\times 10^{-3}$	$7.0\times 10^{-4}$

**Table 2:** Comparison of AAA detection performance metrics for the deep-learning algorithm using arterial blood pressure waveforms (DL-ABP) versus pulse volume recording waveforms (DL-PVR), evaluated at 50% AAA severity level as labeling threshold. Mean ± STD.

	Accuracy	F1 Score	Sensitivity	Specificity	PPV	NPV
DL-ABP	88.8 ± 0.6%	88.9 ± 0.7%	89.5 ± 2.0%	88.2 ± 2.0%	88.4 ± 1.5%	89.4 ± 1.6%
DL-PVR	85.8 ± 0.7%	85.9 ± 0.8%	86.6 ± 2.6%	84.9 ± 2.0%	85.2 ± 1.4%	86.4 ± 2.0%

**Table 3:** Pearson correlation coefficients between two AAA severity metrics (maximum diameter and volumetric severity index (VSI)) and four representative arterial BP waveform features: carotid–femoral pulse wave velocity (PWV), carotid upstroke index (CUI), carotid area ratio (CAR), and carotid oscillatory ratio (COR). All reported correlations are statistically significant (*p* < 0.05).

	PWV	CUI	CAR	COR
Maximum Diameter	−0.389	0.472	0.143	0.413
VSI	−0.393	0.532	0.160	0.458

## Data Availability

The synthetic data supporting the conclusions of this article will be made available by the authors on request.

## Supplementary Materials

The following supporting information can be downloaded at: https://www.mdpi.com/article/10.3390/s25216678/s1, Table S1: Physiological data of the arterial tree used in this study for a 40 year old subject with nominal parameter values. L: Length. r: internal radius. h: wall thickness. E: Young’s modulus. $R_{0},\;{\;R}_{1}$, and C values describe the 3-element Windkessel model coupled with each terminal branch. Reference [48] is cited in the supplementary materials.

## Abbreviations

The following abbreviations are used in this manuscript:

AAA	Abdominal Aortic Aneurysm
BP	Blood Pressure
CNN	Convolutional Neural Network
CPAR	Continuous Property-Adversarial Regularization
CUI	Carotid Upstroke Index
CAR	Carotid Area Ratio
COR	Carotid Oscillatory Ratio
CT	Computed Tomography
DL	Deep Learning
DL-ABP	DL-enabled algorithm for AAA diagnosis based on arterial BP waveform analysis
DL-PVR	DL-enabled algorithm for AAA diagnosis based on PVR waveform analysis
HR	Heart Rate
IIV	Inter-Individual Variability
MAE	Mean Absolute Error
MRI	Magnetic Resonance Imaging
NPV	Negative Predictive Value
PP	Pulse Pressure
PPV	Positive Predictive Value
PRC	Precision–Recall Curve
PVR	Pulse Volume Recording
PWV	Pulse Wave Velocity
ROC	Receiver Operating Characteristic
RMSE	Root Mean Squared Error
SSV	Sample-to-Sample Variability
USPSTF	US Preventive Services Task Force
VSI	Volumetric Severity Index
