# Supplementary material for: Deep Learning-Enabled Diagnosis of Abdominal Aortic Aneurysm Using Pulse Volume Recording Waveforms: An In Silico Study
Source: Sensors (Basel). 2025 Nov 1;25(21):6678. doi: 10.3390/s25216678 (PMC12609100; doi:10.3390/s25216678)
Supplement: Supplementary file 1 [file sensors-25-06678-s001.zip › sensors-3920164-supplementary.pdf]

S1. Systemic Arterial Circulation Model

Details of the systemic arterial circulation model, governing equations, and parameterization are provided here. Full details are originally presented in [1], and later in [2]. The systemic arterial circulation model consists of 55 uniform and linear transmission line (TL) segments, each of which represents an artery characterized by its geometric, viscous, elastic, and inertial properties (see Table S1), with three-element Windkessel loads on terminal branches. In each artery, the inlet and outlet BP and blood flow are related as follows:

$$P_O = \frac{1+\Gamma}{e^{\gamma l} + \Gamma e^{-\gamma l}} P_I, \qquad Q_O = \frac{1-\Gamma}{e^{\gamma l} - \Gamma e^{-\gamma l}} Q_I \tag{S1}$$

where P_I, P_O, Q_I, and Q_O are inlet blood pressure (BP), outlet BP, inlet blood flow rate, and outlet blood flow rate, all pertaining to the artery, Γ is the reflection coefficient of the artery, γ is the propagation constant of the artery, and l is the length of the artery. Γ is given by:

$$\Gamma = \frac{Z_L - Z_C}{Z_L + Z_C} \tag{S2}$$

where Z_L is the load impedance at the outlet of the artery and Z_C is the characteristic impedance of the artery given by:

$$Z_C = \frac{\rho c_0}{A}(1-\sigma^2)^{-\frac{1}{2}}(1-F_{10})^{-\frac{1}{2}} e^{\frac{j\phi}{2}} \tag{S3}$$

where A is the cross-sectional area of the artery, c_0 is pulse wave velocity, σ is the Poisson ratio of the arterial wall, ϕ is the viscoelastic delay of the arterial wall, and $F_{10} = \frac{2J_1(\alpha j^{1.5})}{\alpha j^{1.5} J_0(\alpha j^{1.5})}$ with J_0 and J_1 being the Bessel functions of orders 0 and 1, and α is a frequency-dependent function of blood viscosity [1]. Z_L of an artery is given by (i) its peripheral load given by a 3-element Windkessel if the artery is terminal (i.e., its outlet is not connected to any downstream arteries), or (ii) the input impedance of the artery (or arteries in the case of bifurcation) connected to its outlet (note that such input impedance includes the impedances of all the downstream arteries connected to the artery (or arteries)). The input impedance Z_I of an artery is given by:

$$Z_I = \frac{P_I}{Q_I} = Z_C \frac{e^{\gamma l} + \Gamma e^{-\gamma l}}{e^{\gamma l} - \Gamma e^{-\gamma l}} \tag{S4}$$

Finally, γ is given by:

$$\gamma = \frac{j\omega}{c_0}(1-\sigma^2)^{\frac{1}{2}}(1-F_{10})^{-\frac{1}{2}} e^{-\frac{j\phi}{2}} \tag{S5}$$

Once the aortic flow waveform (at the outlet of the aortic valve) is provided, the systemic arterial circulation model can compute arterial BP and blood flow waveforms at all 55 arteries.

The load impedance for each arterial segment (Equation (S2)) can be calculated using the numbers reported in Table S1, Equations (S3)-(S5), and the backward recursive algorithm described in [1]. For the terminal branches, the load impedance is equal to $Z_L = R_0 + \frac{1}{\frac{1}{R_1} + j\omega C}$, where R_0, R_1, and C values are listed in Table S1.

All other necessary constants for using the Equations (S1)-(S5) are mentioned below.

$$F_{10} = \frac{2J_1(\alpha j^{1.5})}{\alpha j^{1.5} J_0(\alpha j^{1.5})}, \qquad \alpha = r\sqrt{\frac{\omega}{v}}, \qquad v = \frac{\mu}{\rho}$$

$$c_0 = \sqrt{\frac{Eh}{\rho D}}$$

$$\phi = \phi_0(1 - e^{-k\omega}), \qquad \phi_0 = 1° \times \frac{2\pi}{180°} \times \frac{10h}{2r}$$

$\rho = 1.05 \frac{g}{cm^3}$ is the blood density, $\sigma = 0.5$ is the Poisson's ratio of the arterial wall, ω is the circular frequency, v is the kinematic viscosity of the blood, $\mu = 0.0035\ Pa \cdot s$ is the dynamic viscosity of the blood, k is a constant set to 1. Note that Z_L, Z_C, and γ depend on $\omega = 2\pi f$. In calculating these quantities, we considered $0 \le f \le 128$ Hz at a resolution of $\frac{1}{16}$ Hz.

Age-Dependent Parameterization

The age-dependent formulas for stroke volume, proximal aorta length, arterial compliance, and total peripheral resistance were adapted from [3], and are mentioned below:

1. Stroke Volume

We change the stroke volume by modifying the aortic flow input waveform to the model [2].

$$SV[mL] = 72.7 - 0.253 \times age$$

2. Aorta Length and Diameter

The proximal aorta (No. 1, 2, 10, and 12 in Table S1) lengthens with age, whereas the lengths of other arteries do not change. The diameters of the aorta and carotid artery (No. 1, 2, 3, 5, 10, 11, 12, 13, 25, 27, 29, 31, and 33 in Table S1) increase with age, whereas the diameters of the remaining arteries are not affected by age.

$$L[\%] = 80 + 0.8 \times age, \qquad D[\%] = 120 - 6.611 \times 10^{-3} \times (age - 80)^2$$

3. Stiffness

$$E[\%] = 80.2 - 0.349 \times age + 0.0456 \times age^2$$

4. Terminal Compliance

$$C[\%] = 128.4 - 1.136 \times age$$

5. Terminal Resistance R_0 & R_1

$$R[\%] = 78 + 0.51 \times (age - 25)$$

S2. BP-PVR Model

Details of the BP-PVR model, governing equations, and parameterization are provided here. Full details are originally presented in [4], and later in [5].

The transfer functions governing the viscoelastic BP-PVR relationships at the brachial and tibial sites are given by:

$$Y_B(s) = \frac{E_{B2} + \eta_B s}{E_{B1}E_{B2} + (E_{B1} + E_{B2})\eta_B s} P_B(s), \qquad Y_T(s) = \frac{1}{E_T + \eta_T s} P_T(s) \tag{S6}$$

where E_{B1}, E_{B2} and η_B are sampled as below:

$$z_1 \sim \mathcal{N}(0, 0.453^2), \qquad z_2 = y - 0.425, \quad y \sim \text{Rayleigh}(0.347), \qquad z_3 \sim \mathcal{N}(0, 0.40^2)$$

$$\begin{bmatrix} E_{B1} \\ E_{B2} \\ \eta_B \end{bmatrix} = \begin{bmatrix} -0.588 & 0.722 & 0.365 \\ 0.782 & 0.623 & 0.026 \\ 0.209 & -0.300 & 0.931 \end{bmatrix} \begin{bmatrix} z_1 \\ z_2 \\ z_3 \end{bmatrix} + \begin{bmatrix} 0.491 \\ 1.482 \\ 0.223 \end{bmatrix}$$

while E_T and η_T are sampled as below:

$$z_1 \sim \mathcal{N}(0, 0.085^2), \qquad z_2 \sim \mathcal{N}(0, 0.015^2)$$

$$\begin{bmatrix} E_T \\ \eta_T \end{bmatrix} = \begin{bmatrix} 0.920 & 0.076 \\ -0.076 & 0.997 \end{bmatrix} \begin{bmatrix} z_1 \\ z_2 \end{bmatrix} + \begin{bmatrix} 1.022 \\ 0.026 \end{bmatrix}$$

Feasibility and stability constraints (based on which irrelevant samples are excluded) include: (i) $E_{B1}, E_{B2}, \eta_B, E_T, \eta_T > 0$, (ii) $E_{B1} \times E_{B2} > 2f_s\eta_B(E_{B1} + E_{B2})$, and (iii) $E_T > 2f_s\eta_T$.

Table S1: Physiological data of the arterial tree used in this study for a 40 year old subject with nominal parameter values. L: Length. r: internal radius. h: wall thickness. E: Young's modulus. R_0, R_1, and C values describe the 3-element Windkessel model coupled with each terminal branch.

No.	Arterial Segment	L (cm)	r (cm)	h (cm)	E (Mpa)	R_0 ($10^9 Pa.s/m^3$)	R_1 ($10^9 Pa.s/m^3$)	C ($10^{-10} m^3/Pa$)
1	Ascending aorta	2.016	1.856	0.163	0.724	—	—	—
2	Aortic arch I	3.024	1.736	0.126	0.724	—	—	—
3	Brachiocephalic	3.15	0.799	0.08	0.724	—	—	—
4	R. subclavian I	3.15	0.483	0.067	0.724	—	—	—
5	R. carotid	15.93	0.484	0.063	0.724	—	—	—
6	R. vertebral	12.15	0.23	0.045	1.447	1.421	6.493	1.694
7	R. subclavian II	35.82	0.368	0.067	0.724	—	—	—
8	R. radius	19.8	0.184	0.043	1.447	3.311	4.273	1.922
9	R. ulnar I	6.03	0.253	0.046	1.447	—	—	—
10	Aortic arch II	4.032	1.629	0.115	0.724	—	—	—
11	L. carotid	18.72	0.484	0.063	0.724	—	—	—
12	Thoracic aorta I	5.544	1.491	0.11	0.724	—	—	—
13	Thoracic aorta II	9.45	1.283	0.11	0.724	—	—	—
14	Intercostals	6.57	0.345	0.049	0.724	0.466	1.407	7.285
15	L. subclavian I	3.15	0.483	0.066	0.724	—	—	—
16	L. vertebral	12.15	0.23	0.045	1.447	1.421	6.493	1.694
17	L. subclavian II	35.82	0.368	0.067	0.724	—	—	—
18	L. ulnar I	6.03	0.253	0.046	1.447	—	—	—
19	L. radius	19.8	0.184	0.043	1.447	3.311	4.273	1.922
20	Celiac I	1.8	0.374	0.064	0.724	—	—	—
21	Celiac II	1.8	0.316	0.064	0.724	—	—	—
22	Hepatic	5.85	0.305	0.049	0.724	0.652	4.073	2.796
23	Splenic	5.22	0.19	0.054	0.724	2.001	5.352	1.868
24	Gastric	4.95	0.23	0.045	0.724	0.944	2.234	4.369
25	Abdominal aorta I	4.77	1.107	0.09	0.724	—	—	—
26	Sup. mesenteric	4.5	0.431	0.069	0.724	0.28	0.967	10.888
27	Abdominal aorta II	1.35	1.038	0.08	0.724	—	—	—
28	R. renal	2.7	0.322	0.053	0.724	0.471	1.081	8.966
29	Abdominal aorta III	1.35	1.007	0.08	0.724	—	—	—
30	L. renal	2.7	0.322	0.053	0.724	0.471	1.081	8.966

31	Abdominal aorta IV	11.25	0.893	0.075	0.724	—	—	—
32	Inf. mesenteric	3.42	0.218	0.043	0.724	1.251	7.712	1.479
33	Abdominal aorta V	7.2	0.742	0.065	0.724	—	—	—
34	R. com. iliac	5.22	0.443	0.06	0.724	—	—	—
35	R. ext. iliac	13.05	0.391	0.053	1.447	—	—	—
36	R. int. iliac	4.05	0.23	0.04	2.895	1.491	5.89	1.828
37	R. deep femoral	10.17	0.23	0.047	1.447	1.163	3.355	3.038
38	R. femoral	39.87	0.339	0.05	1.447	—	—	—
39	R. ext. carotid	15.93	0.23	0.042	1.447	1.219	5.482	1.989
40	L. int. carotid	15.84	0.333	0.045	1.447	0.589	5.922	1.989
41	R. post. tibial	30.96	0.207	0.045	2.895	2.307	7.637	1.371
42	R. ant. tibial	28.98	0.287	0.039	2.895	0.923	3.52	3.038
43	R. interosseous	6.3	0.115	0.028	2.895	9.187	98.788	0.121
44	R. ulnar II	15.3	0.218	0.046	1.447	2.358	4.939	1.922
45	L. ulnar II	15.3	0.218	0.046	1.447	2.358	4.939	1.922
46	L. interosseous	6.3	0.115	0.028	2.895	9.187	98.788	0.121
47	R. int. carotid	15.84	0.333	0.045	1.447	0.589	5.922	1.989
48	L. ext. carotid	15.93	0.23	0.042	1.447	1.219	5.482	1.989
49	L. com. iliac	5.22	0.443	0.06	0.724	—	—	—
50	L. ext. iliac	13.05	0.391	0.053	1.447	—	—	—
51	L. int. iliac	4.05	0.23	0.04	2.895	1.491	5.89	1.828
52	L. deep femoral	10.17	0.23	0.047	1.447	1.163	3.355	3.038
53	L. femoral	39.87	0.339	0.05	1.447	—	—	—
54	L. post. tibial	28.98	0.207	0.045	2.895	2.307	7.637	1.371
55	L. ant. tibial	30.96	0.287	0.039	2.895	0.923	3.52	3.038

References

1. HE, W.; XIAO, H.; LIU, X. NUMERICAL SIMULATION OF HUMAN SYSTEMIC ARTERIAL HEMODYNAMICS BASED ON A TRANSMISSION LINE MODEL AND RECURSIVE ALGORITHM. *J Mech Med Biol* 2012, *12*, 1250020, doi:10.1142/S0219519411004587.
2. Shahrbabak, S.M.; Mousavi, A.; Mukkamala, R.; Hahn, J.O. In Silico Investigation of a Mathematical Model Relating the Ballistocardiogram to Aortic Blood Pressure. *IEEE Trans Biomed Eng* 2025, doi:10.1109/TBME.2025.3584979.
3. Charlton, P.H.; Mariscal Harana, J.; Vennin, S.; Li, Y.; Chowienczyk, P.; Alastruey, J. Modeling Arterial Pulse Waves in Healthy Aging: A Database for in Silico Evaluation of Hemodynamics and Pulse Wave Indexes. *American Journal of Physiology-Heart and Circulatory Physiology* 2019, *317*, 1062–1085, doi:10.1152/ajpheart.00218.2019.
4. Lee, J.; Ghasemi, Z.; Kim, C.S.; Cheng, H.M.; Chen, C.H.; Sung, S.H.; Mukkamala, R.; Hahn, J.O. Investigation of Viscoelasticity in the Relationship between Carotid Artery Blood Pressure and Distal Pulse Volume Waveforms. *IEEE J Biomed Health Inform* 2018, *22*, 460–470, doi:10.1109/JBHI.2017.2672899.
5. Masoumi Shahrbabak, S.; Kim, S.; Youn, B.D.; Cheng, H.M.; Chen, C.H.; Mukkamala, R.; Hahn, J.O. Peripheral Artery Disease Diagnosis Based on Deep Learning-Enabled Analysis of Non-Invasive Arterial Pulse Waveforms. *Comput Biol Med* 2024, *168*, 107813, doi:10.1016/J.COMPBIOMED.2023.107813.
